# Neonatal-onset cyanotic breath-holding spells: a case report with longitudinal diagnostic dilemma

**DOI:** 10.3389/fped.2026.1783586

**Published:** 2026-04-07

**Authors:** Abdulaziz Alosail, Amal Alotaibi, Rawan Jarwann, Abdullah AlHojailan, Meshal AlRashed, Abdulmajeed AlFadhel, Tamer Abusido

**Affiliations:** 1 Department of Pediatrics, Ministry of National Guard Health Affairs, King Abdullah Specialized Children Hospital, King Abdulaziz Medical City, Riyadh, Saudi Arabia; 2 King Saud bin Abdulaziz University for Health Sciences, Riyadh, Saudi Arabia; 3 King Abdullah International Medical Research Centre, Riyadh, Saudi Arabia

**Keywords:** breath holding, crying, cyanotic spells, neonatal hypoxemia, spells

## Abstract

Breath-holding spells (BHS) are benign paroxysmal events that typically present between 6 and 18 months of age. Neonatal-onset BHS is rare and may closely mimic other causes of neonatal cyanotic episodes, particularly respiratory inhibition after crying (RIAC). We report a term male infant who developed recurrent cyanotic episodes starting at birth and underwent extensive investigations with unremarkable results. He was initially diagnosed with prolonged RIAC. Longitudinal follow-up demonstrated persistence of episodes with evolution into a classical cyanotic breath-holding phenotype. This case highlights the importance of longitudinal assessment in neonates with unexplained cyanotic spells and supports the inclusion of breath-holding spells in the differential diagnosis after exclusion of serious underlying conditions.

## Introduction

Breath-holding spells occur in approximately 0.1%–4.6% of otherwise healthy children and most commonly begin between 6 and 18 months of age (1). Two clinical subtypes are recognized: cyanotic and pallid. Episodes are commonly precipitated by emotional distress or pain and may be followed by transient loss of consciousness or brief convulsive movements secondary to cerebral hypoxia (1).

Presentation before six months of age is uncommon, and neonatal-onset BHS is rarely reported (8). In the neonatal period, respiratory inhibition after crying (RIAC) represents an important diagnostic consideration, as it presents with prolonged hypoxemia following crying and typically resolves within the first month of life. Distinguishing between these entities can be challenging early in life and often requires longitudinal observation (2).

## Case presentation

A term male infant was born at 41 weeks’ gestation via cesarean section due to non-reassuring cardiotocography. Birth weight was 3,540 g, with APGAR scores of 5 and 8 at one and five minutes, respectively. Antenatal history was unremarkable, with no maternal illness, perinatal infection, or substance exposure. Initial neonatal physical examination was normal.

Within the first hours of life, the infant developed recurrent episodes of central cyanosis occurring predominantly during crying. These episodes were characterized by breath holding and oxygen desaturation to approximately 70%, without associated bradycardia. During the first week of life, episodes occurred up to five times daily, prompting admission to the neonatal intensive care unit for further evaluation and monitoring. During typical episodes, the infant initially developed vigorous crying followed by apparent breath-holding during expiration. Cyanosis developed shortly thereafter, accompanied by oxygen desaturation. Episodes generally lasted several seconds to approximately one minute and resolved spontaneously once breathing resumed. Recovery was rapid, and the infant returned to baseline behavior shortly after the event without postictal symptoms or prolonged lethargy.

Throughout his 40-day neonatal intensive care unit stay, the infant remained hemodynamically stable between episodes and did not require respiratory support or supplemental oxygen outside of acute events. Feeding was well tolerated, with no choking, vomiting, or aspiration. No abnormal movements, eye deviation, or postictal features were observed during or after the episodes.

**Figure 1:**
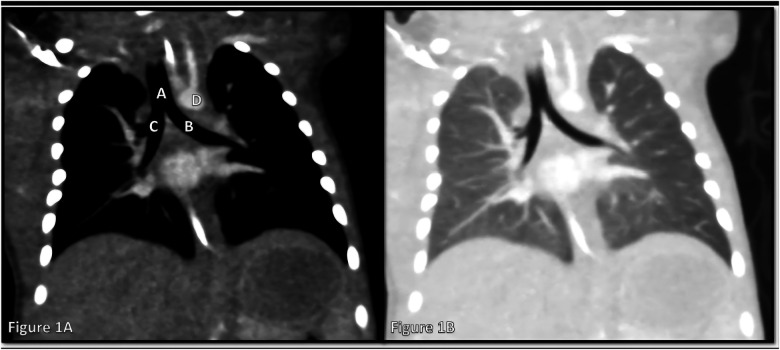
Coronal chest CT images demonstrating normal central airway anatomy and vascular relationships. **(A)** Lung window view showing the trachea **(A)**, right main bronchus **(B)**, left main bronchus **(C)**, and the aortic arch **(D)** coursing over the left main bronchus without evidence of airway compression. **(B)** Corresponding coronal reconstruction highlighting normal lung parenchyma and airway branching.

**Figure 2:**
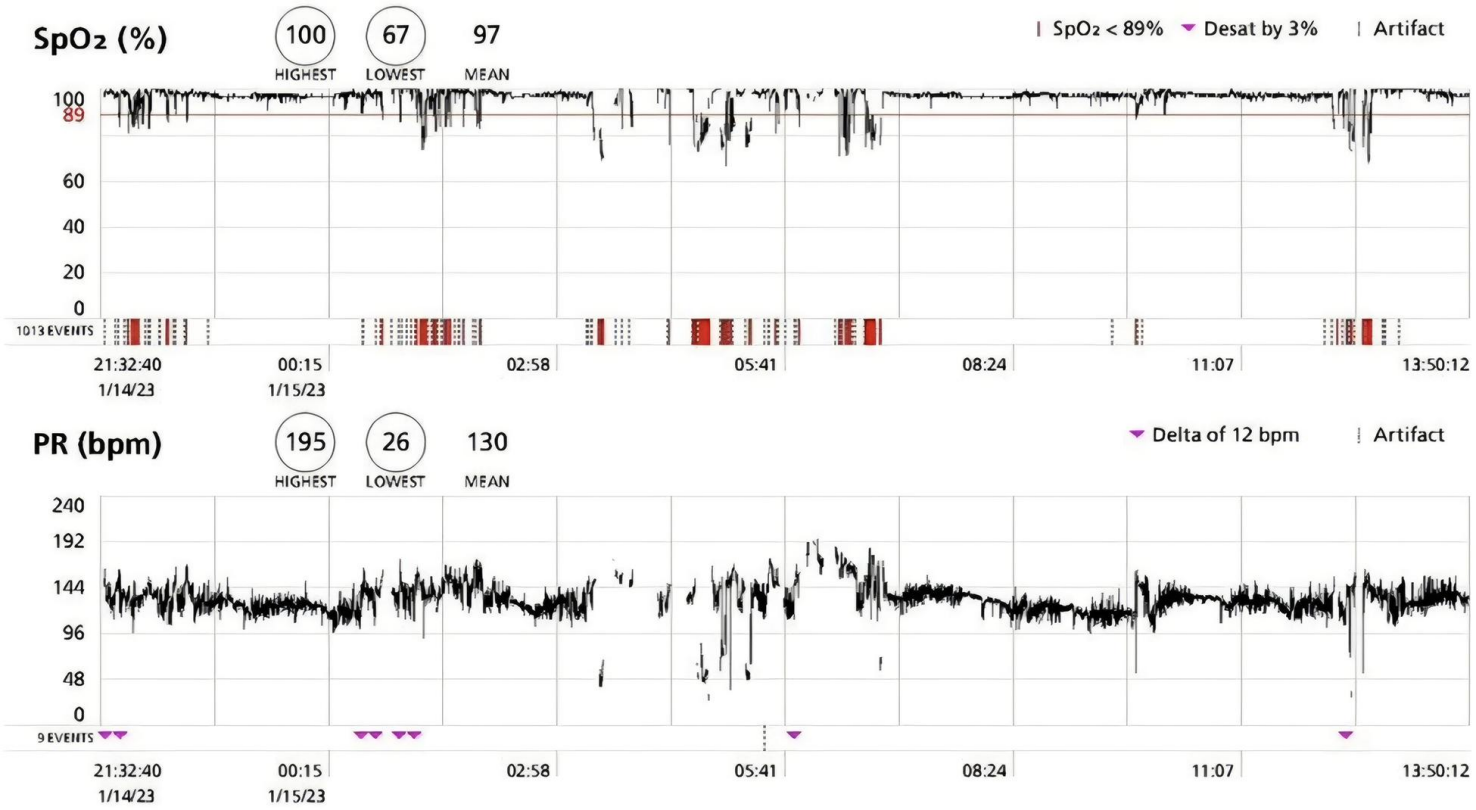
Overnight pulse oximetry demonstrating recurrent clusters of desaturation temporally associated with crying episodes.

An extensive diagnostic evaluation was undertaken to investigate potential causes of neonatal cyanosis. Cardiac assessment, including echocardiography, revealed no structural or functional abnormalities except for a small Patent foramen ovale that required only Follow up. Electrocardiography was performed to evaluate for potential cardiac arrhythmias, including long QT syndrome, which is an important condition to exclude in infants presenting with paroxysmal cyanotic events and was normal for age. Moreover, laboratory evaluation including hemoglobin and iron studies did not demonstrate evidence of anemia or iron deficiency. Neurological evaluation and electroencephalography showed no evidence of epileptic activity during the episodes. Chest imaging and extensive airway assessment, including chest radiography (CXR), computed tomography (CT), and bronchoscopy, did not identify anatomical abnormalities (Figure 1). Overnight pulse oximetry with nursing documentation demonstrated that the episodes were not related to sleep and occurred predominantly during agitation or crying, followed by breath-holding (Figure 2). Neurometabolic workup, including brain MRI and basic metabolic screening, was unremarkable, as was infectious workup (Table 1).

**Table 1:** Summary of workup.

System evaluated	Type of study	Result
Cardiac	Echocardiography ECG	Normal cardiac structure and function
Neurological	MRI brain Electroencephalography (EEG)	No epileptic activity
Respiratory	CXR CT Bronchoscopy	No anatomical abnormalities
Metabolic	Metabolic screening	Unremarkable
Infectious	Sepsis/infectious workup	Unremarkable
Gastrointestinal	Swallowing, UGI, Milk scan	Unremarkable

Given the temporal association of episodes with crying and the absence of an identifiable etiology, the episodes were initially attributed to prolonged respiratory inhibition after crying. Management was conservative and focused on supportive measures, including minimizing prolonged crying, optimizing feeding comfort, and encouraging maternal presence, which appeared to reduce episode frequency. Upon discharge, empirical proton pump inhibitor therapy was initiated, and the family was educated regarding reflux precautions.

The infant was discharged home with close outpatient follow-up by neonatology and pediatric pulmonology teams. After discharge, the frequency and characteristics of the episodes were primarily documented based on parental observations reported during scheduled follow-up visits. Over time, the frequency of cyanotic episodes gradually decreased. During infancy and early toddlerhood, caregivers noted that episodes became increasingly associated with emotional triggers, particularly frustration or anger. By 28 months of age, the events had evolved into a classical cyanotic breath-holding pattern, occurring only once every few months and exclusively with vigorous crying. Between episodes, the child remained asymptomatic.

**Figure 3:**
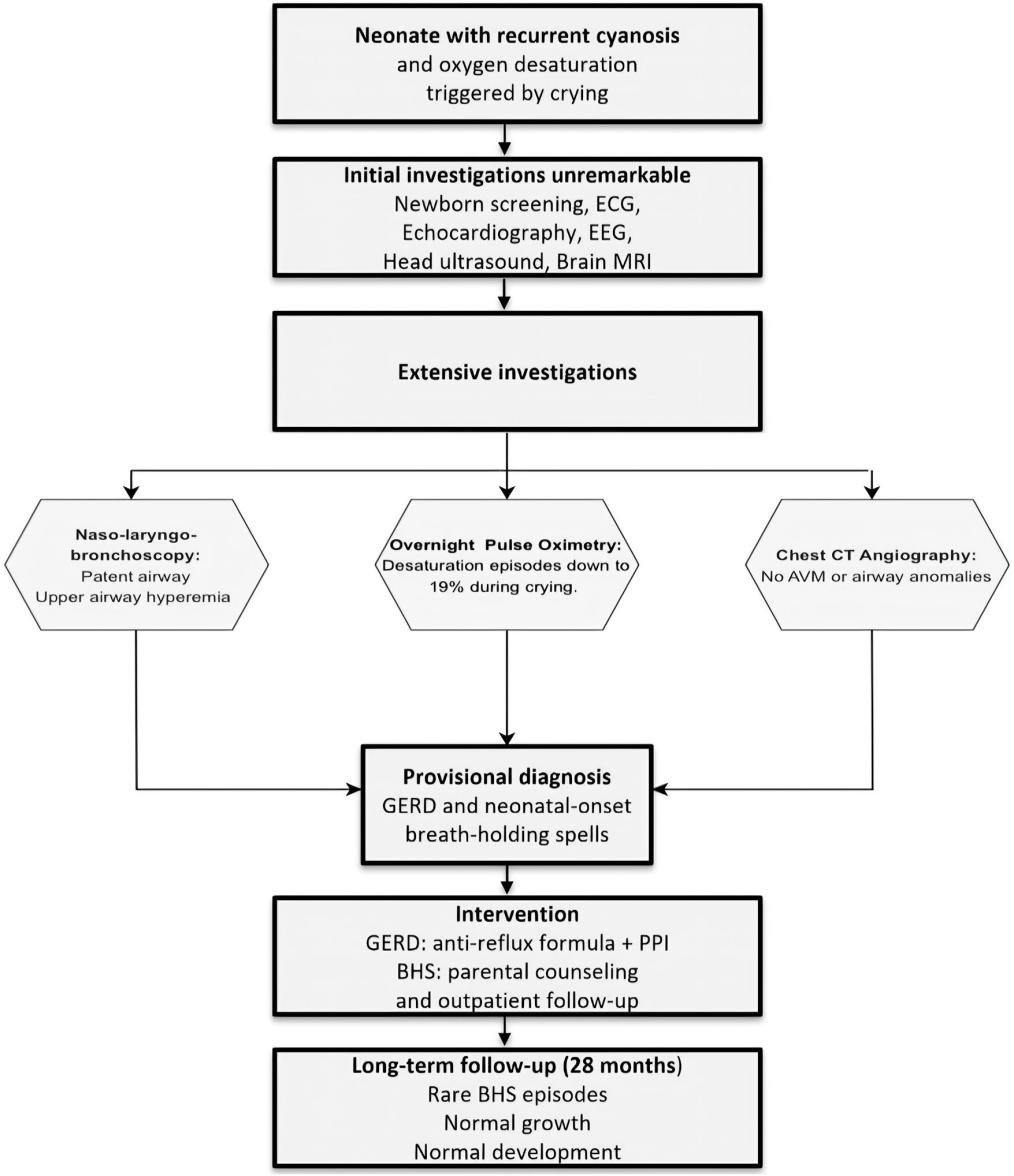
Flowchart illustrating the diagnostic and management journey of our patient from day one till last follow up.

Throughout longitudinal follow-up, the patient demonstrated normal growth, age-appropriate neurodevelopment, and no behavioral abnormalities. No new neurological or cardiopulmonary symptoms emerged, supporting the revised diagnosis of neonatal-onset cyanotic breath-holding spells (Figure 3).

## Discussion

This case illustrates a rare presentation of cyanotic breath-holding spells beginning in the neonatal period and highlights the diagnostic challenges encountered when evaluating recurrent neonatal cyanotic episodes. Breath-holding spells are typically diagnosed clinically in older infants and toddlers; however, neonatal onset is uncommon and may closely resemble other causes of neonatal hypoxemia (3). The diagnosis of breath-holding spells is primarily clinical and relies on recognition of the characteristic sequence of events, as universally accepted diagnostic criteria are lacking. In our patient, early presentation, severity of desaturation, and prolonged neonatal course supported an initial diagnosis of respiratory inhibition after crying. Respiratory inhibition after crying has been primarily described by a limited number of investigators, particularly Minowa and colleagues. Although RIAC is not universally recognized as a distinct clinical entity across all pediatric centers, its clinical presentation overlaps with neonatal cyanotic events and therefore remains relevant in the differential diagnosis. RIAC episodes typically begin with vigorous crying followed by breath-holding during expiration, subsequent cyanosis, and spontaneous recovery once breathing resumes. It has been proposed to result from delayed maturation of respiratory control and associated with adverse neurodevelopmental outcomes. In contrast, our patient demonstrated normal growth, neurodevelopment, and behavior, arguing against this diagnosis (7). An etiological hypothesis of impaired maturation of the respiratory center due to *in utero* stress has been implicated. It encompasses three primary forms: respiratory inhibition after crying (RIAC), feeding-related hypoxemia, and respiratory inhibition following gastroesophageal reflux (RIGER). The prognosis of neonatal respiratory inhibition includes short stature, delayed motor and speech development, and significant behavioural changes, such as stereotypic interests or behaviours, or hyperactivity (7). Initially, our probable differential diagnosis was prolonged neonatal inhibition after crying; however, longitudinal follow-up proved essential in clarifying the diagnosis. Persistence of episodes beyond infancy, along with the emergence of a classical emotional trigger and a marked reduction in frequency, supported reclassification as neonatal-onset cyanotic breath-holding spells (5). This diagnostic evolution underscores the limitations of cross-sectional assessment during the neonatal period.

Neonatal onset of BHS is rare; however, a few cases have been reported in the literature. Familial clustering has been described, with a positive family history reported in up to one-third of affected children. Although this suggests a possible genetic predisposition, the underlying mechanisms remain poorly understood. Associations with conditions involving autonomic dysfunction further support a role for autonomic immaturity in the pathophysiology of early-onset breath-holding spells (5). Another case was reported in 2008 of a neonate presenting with BHS from the age of 2 days of life; in contrast, this case had no familial predisposition (6, 9).

Approximately 23%–38% of patients reported a positive family history of breath-holding spells (3). Nevertheless, the exact relationship between this observation and the BHS mechanism has not been identified. A case series in Australia reported familial cyanotic BHS in seven siblings, some with neonatal onset. All siblings underwent investigations and had normal cardiac, neurological, and metabolic profiles. Five of the siblings were prescribed an antiepileptic drug, which resulted in a significant decrease in breath-holding episodes (3). Another study found an increased incidence of BHS in patients with familial dysautonomia, reaching 53% compared with 4%–21% in the general population (4). It is important to note that most children with breath-holding spells are otherwise healthy and do not have underlying genetic or neurological disorders (1).

Finally, breath-holding spells remain a diagnosis of exclusion in neonates. Serious conditions, including epilepsy, congenital heart disease, structural airway abnormalities, gastroesophageal reflux–related laryngospasm, and inborn errors of metabolism, must be carefully excluded before establishing the diagnosis. Recognition of this benign and self-limited condition can prevent unnecessary investigations, inappropriate pharmacological treatment, and significant parental anxiety.

## Key learning points

- Neonatal-onset cyanotic breath-holding spells are rare and may mimic other causes of neonatal hypoxemia.
- Respiratory inhibition after crying and early-onset breath-holding spells can be difficult to distinguish in the neonatal period.
- Longitudinal clinical follow-up is essential for accurate diagnosis when initial investigations are unrevealing.
- Breath-holding spells are a diagnosis of exclusion in neonates and require thorough evaluation to rule out serious pathology.
- Early recognition allows appropriate parental reassurance and conservative management.

## Conclusion

This case report highlights a rare neonatal presentation of cyanotic breath-holding spells. When a newborn presents with recurrent cyanotic events, serious differential diagnoses must be carefully excluded. In our patient, extensive evaluation yielded unremarkable results, and longitudinal follow-up supported the diagnosis of neonatal-onset breath-holding spells. Recognition of this benign and self-limited condition can help clinicians provide appropriate counselling, avoid overtreatment, and reduce parental anxiety until spontaneous resolution in early childhood.

## Data Availability

The original contributions presented in the study are included in the article/Supplementary Material, further inquiries can be directed to the corresponding author.
